# Silver Nanowires Epsilon‐Negative Metacomposites in Constructing Laminated Structure Meta‐Capacitors

**DOI:** 10.1002/smll.202501848

**Published:** 2025-06-30

**Authors:** Zongxiang Wang, Kai Sun, Yuan Yuan, Xin Yao, Qing Hou, Chaoyun Song, Runhua Fan

**Affiliations:** ^1^ Logistics Engineering College Shanghai Maritime University Shanghai 201306 China; ^2^ College of Ocean Science and Engineering Shanghai Maritime University Shanghai 201306 China; ^3^ Department of Engineering Faculty of Natural Mathematical & Engineering Sciences King's College London London WC2R 2LS UK; ^4^ Shandong Key Laboratory of Metamaterial and Electromagnetic Manipulation Technology Jinan 250061 China

**Keywords:** epsilon‐negative materials, laminated meta‐capacitors, metacomposites, percolation structures, plasma oscillation

## Abstract

Epsilon‐negative metacomposites with remarkable super‐coupling, tunneling, and local field enhancements characteristics have attracted extensive attention. Under the growing demand for superior energy storage and conversion efficiency of capacitors, conventional materials can not fulfill the requirements in balancing the trade‐offs of dielectric constant, losses, and energy density. New insights into the design and functionality of laminated structure meta‐capacitors are presented, which can provide an effective solution to these limitations. The proposed meta‐capacitors are made of polyvinylidene fluoride (PVDF), barium titanate (BaTiO_3_), and silver nanowires (AgNWs), which exhibit excellent dielectric properties and higher stored/discharged energy density. Specifically, epsilon‐negative behavior is derived from plasma oscillation via constructing percolation networks. Consequently, the dielectric constant of meta‐capacitors is significantly enhanced to 59.74, which is more than six times that of pristine PVDF of 9.95, while the loss tangent is lower than 0.035. Notably, compared with conventional devices, the meta‐capacitors have shown higher energy storage and discharge density, which is increased by 148% and 133% respectively. This work highlights the potential of laminated structure meta‐capacitors in promising energy applications, which can provide valuable insights for next‐generation energy storage devices.

## Graphical abstract

The enhancement of positive dielectric constant value from 8.22 to 29.44 is attributed to the interfacial polarization, and the negative dielectric constant value of −8.74 is due to plasma oscillation stemming from conductive AgNWs networks. Moreover, epsilon‐negative and epsilon‐positive materials are explored to fabricate laminated structure meta‐capacitors.

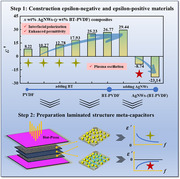

## 1 Introduction

The extension of the dielectric constant from positive to negative provides new insights into dielectric parameters, enriching the field of dielectrics.^[^ ^1^, ^2^ ^]^ Particularly noteworthy is a remarkable category within metacomposites that has recently seen booming success.^[^ ^3^, ^4^ ^]^ This success is largely due to their supercoupling, tunneling effects, and local field enhancement capabilities. Since their introduction, the study of epsilon‐negative properties has attracted tremendous interest, both fundamental and practical, in the realm of electrical energy. At present, the escalating challenges posed by fossil fuel consumption and global climate change underscore the need for solutions that enhance energy storage and conversion efficiency. In the realm of electrical energy applications, conversion efficiency is undergoing exponential improvements, paralleling Moore's law.^[^ ^2^ ^]^ Unlike the epsilon‐positive property, which reflects a material's polarization potential, the epsilon‐negative property arises from the motion of free charge carriers. This movement is crucial for the strong local field enhancement effect that is essential for high‐performance capacitors.

Currently, polymer‐based composites filled with conductive fillers are considered a promising approach to enhance energy storage and conversion efficiency, offering high dielectric constant, high energy density, and low losses.^[^ ^5^, ^6^ ^]^ It is typically assumed that improvement of dielectric properties is always attributed to constructing capacitive circuit networks. Under an electric field, capacitors derived from conducting fillers exhibit a strong ability in the polarization process, resulting in a substantial increase in the dielectric constant.^[^ ^7^, ^8^ ^]^ Based on the above insights, fillers such as metal particles and nanowires and nanosheets, as well as various polymer‐based matrices, have been extensively studied in the field of capacitors.^[^ ^9^, ^10^ ^]^ Compared with the dielectric constant of pristine epoxy of 3.6, W‐WO_3_/BaTiO_3_/epoxy composites demonstrated a high dielectric constant of 536, and low dielectric losses of 0.05.^[^ ^11^ ^]^ Additionally, the size of conducting fillers also played a crucial role in dielectric performances. For instance, the dielectric constant of Ti_3_C_2_T_x_ flakes (ca. 4.5 µm) was more than 10 times higher than that of Ti_3_C_2_T_x_ flakes (ca. 1.5 µm).^[^ ^12^ ^]^ Among polymer‐based matrices, PVDF exhibited a relatively high dielectric constant of 10, significantly larger than that of polystyrene of 2.6, epoxy of 3.6, and polyimide of 4.0.^[^ ^11^, ^13^, ^14^ ^]^ It should be noted that an increase in dielectric performance always accompanies with higher content of conducting fillers, resulting in a detrimental to breakdown strength.^[^ ^8^ ^]^ Therefore, laminated composites with “soft‐hard‐soft” or “hard‐soft‐hard” structures have gained considerable attention in novel capacitors. Multilayer composites have demonstrated the potential to overcome the traditional trade‐off between dielectric constant and dielectric loss by combining the advantages of hard and soft layers, where the hard layer provides high breakdown strength, and the soft layer contributes a higher dielectric constant. For instance, BaTiO₃/P(VDF‐HFP) composites with a gradient‐layered structure achieved a high dielectric constant of 10.7, representing a 20% improvement over the P(VDF‐HFP) matrix, while maintaining a low loss tangent of 0.05.^[^ ^15^ ^]^ Similarly, sandwich‐structured Ba(Fe₀.₅Ta₀.₅)O₃/PVDF‐Ni/PVDF composites exhibited a high permittivity of ≈130 with relatively low dielectric loss of 0.14 at 1 kHz.^[^ ^16^ ^]^ Despite these advancements, a fundamental challenge remains: achieving both high permittivity and low dielectric loss simultaneously. This underscores the need for innovative dielectric material designs capable of meeting the stringent performance requirements of practical capacitor applications.

As reported, the soft layer or hard layer is mainly composed of epsilon‐positive composites, while epsilon‐negative composites are rarely studied. To our best knowledge, epsilon‐negative metacomposites possess strong charge accumulation ability and local field enhancement effect that will make a superb contribution to dielectric performances.^[^ ^17^ ^]^ For the concept of meta‐capacitors, from the perspective of material structure, a meta‐capacitor was composed of three layers, including an epsilon‐positive layer/an epsilon‐negative layer/epsilon‐positive layer. From the internal mechanism, the unique property of epsilon‐negative behavior derived from plasma oscillation was explored to prepare the meta‐capacitors, in which it can generate strong interfacial polarization and localized electric field enhancement effect, leading to the improvement of dielectric performance and energy storage of capacitors. According to the effective medium theory, the equivalent capacitance will be smaller than that of any individual capacitor. If the equivalent capacitance of the middle layer becomes negative and its absolute value is comparable to the equivalent positive capacitances of the outer layers, an infinite capacitance as well as an infinite dielectric constant can be achieved.^[^ ^18^ ^]^ Hence, epsilon‐negative composites can be regarded as the soft layer, displaying robust polarization capabilities when positioned as the middle layer within laminated structure meta‐capacitors. Introducing an epsilon‐negative layer into meta‐capacitors characterized by a higher dielectric constant and energy density represents a relatively innovative design concept.

Hence, we conducted a comprehensive investigation to elucidate the underlying mechanisms of epsilon‐negative composites and energy storage performances. Epsilon‐negative behavior was derived from plasma oscillation and can be tuned by controlling the distribution of AgNWs, and those materials were used as a middle layer to prepare laminated structure meta‐capacitors. Consequently, a store energy density of 0.52 J cm^−3^ and a discharge energy density of 0.42 J cm^−3,^ and a higher dielectric constant of 59.74 and lower losses of 0.035 were achieved. These findings provide valuable insights for the design of similar laminated structure meta‐capacitors and underscore their promising applications for next‐generation capacitors in the field of electric energy.

## 2 Research and Discussion

**Figure 1:**
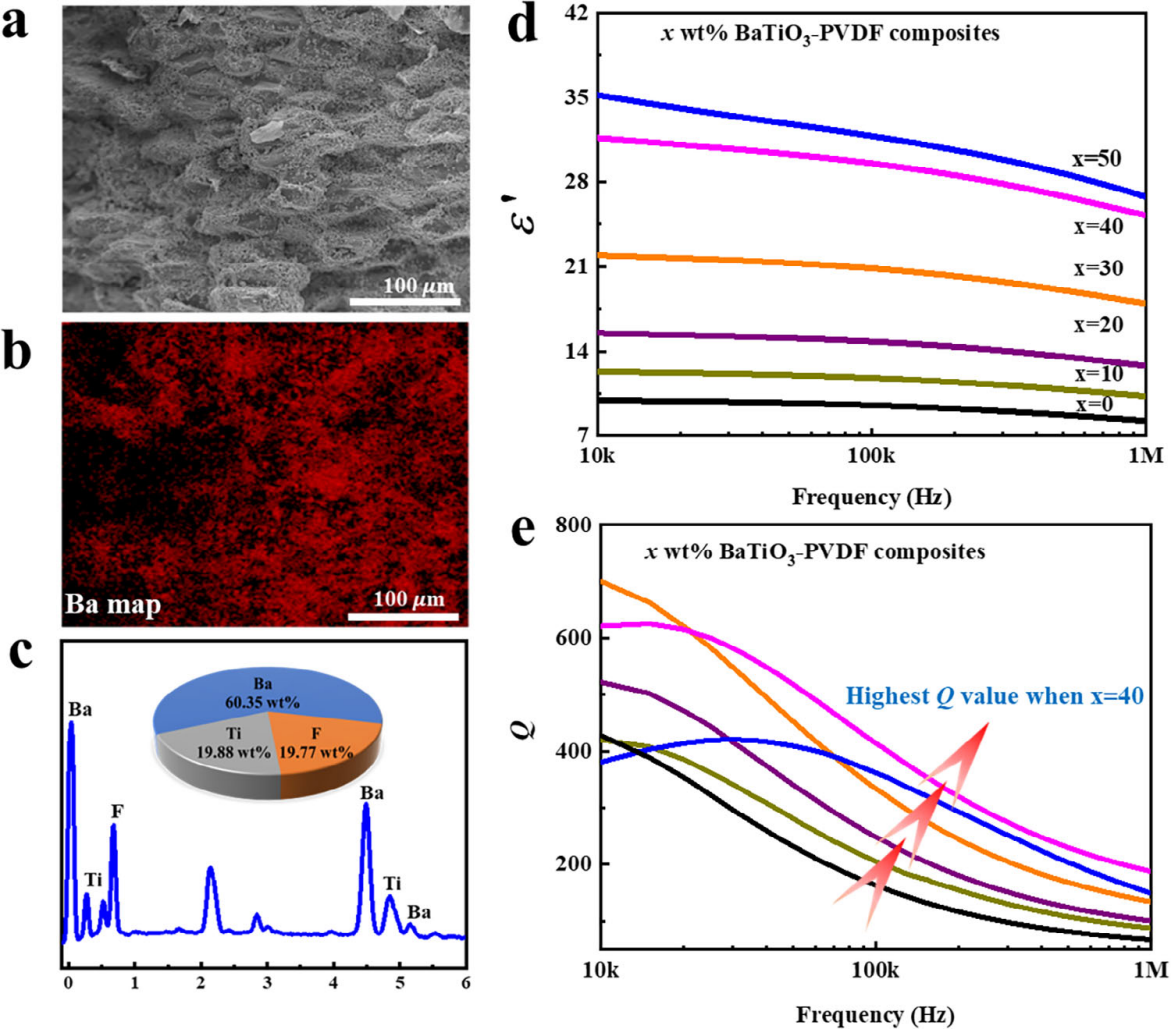
SEM image a) and Ba map b) and EDS spectra c) of 40 wt% BaTiO3‐PVDF composites, dielectric constant d) and Q value spectrum e) of x wt% BaTiO3‐PVDF composites.

Microstructures and dielectric properties of *x* wt% BaTiO_3_‐PVDF composites are shown in **Figure** **1**. Typical SEM images and EDS maps of 40 wt% BaTiO_3_‐PVDF composites are shown in Figure 1a–c, BaTiO_3_ particles are uniformly distributed in the PVDF matrix with a slight agglomeration. A Ba map of 40 wt% BaTiO_3_‐PVDF composites clearly illustrates changes in elemental composition, and characteristic elements of Ba, Ti, and F are attributed to BaTiO_3_ and PVDF materials. The influence of BaTiO_3_ content on dielectric properties of *x* wt% BaTiO_3_‐PVDF composites is illustrated in Figure 1d,e. In Figure 1d, it is evident that the dielectric constant increases as BaTiO_3_ content increases. A slight decrease with increasing frequency is attributed to the dielectric relaxation.^[^ ^19^, ^20^ ^]^ When the BaTiO_3_ content is increased to 50 wt%, a considerable enhancement of the dielectric constant of 35.2 is 3.5 times larger than that of pristine PVDF of 9.9. Theoretically, the increase of dielectric constant is attributed to micro‐capacitor networks derived from BaTiO_3_, resulting in stronger interfacial polarization.^[^ ^21^, ^22^ ^]^ The *Q* value (*Q* = ɛ′ /tanθ, see Figure 1e) is used to evaluate the dielectric performance, the higher *Q* values indicate higher dielectric constant and low losses. Among those materials, 40 wt% BaTiO_3_‐PVDF composites display the highest *Q* value, making it a suitable matrix in achieving epsilon‐negative composites.

**Figure 2:**
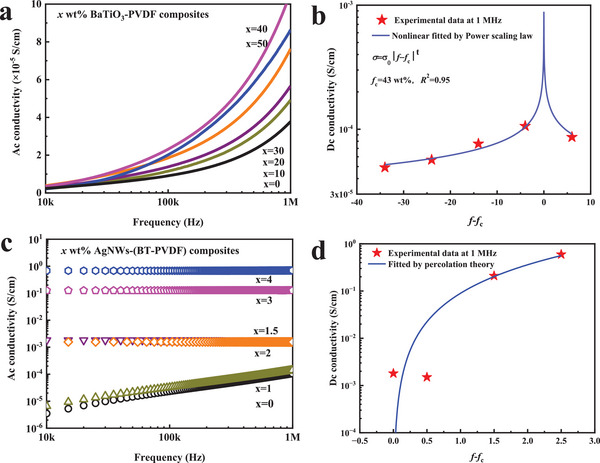
Frequency dispersion of Ac conductivity a, c) and Dc conductivity b, d) of *x* wt% BaTiO_3_‐PVDF composites and *x* wt% AgNWs‐(BT‐PVDF) composites.

Frequency dispersion of Ac conductivity and Dc conductivity of BaTiO_3_‐PVDF and AgNWs‐(BT‐PVDF) composites are displayed in **Figure** **2**. Ac conductivity values increase with both increasing BaTiO_3_ mass fraction and frequency, as shown in Figure 2a. Ac conductivity behavior follows a typical dielectric performance defined as σ_Ac_∝*f*, indicating classic hopping conduction.^[^ ^18^ ^]^ For this case, the Power scaling law is explored to examine the percolation threshold of *x* wt% BaTiO_3_‐PVDF composites, which is described as σ  = σ_0_ |*f* − *f*_c_|^*t*^ in Figure 2b, σ is Dc conductivity, σ_0_ is scaling factor, *f* and *f*_c_ are mass fraction and percolation threshold of BaTiO_3_, *t* is critical exponent.^[^ ^23^, ^24^ ^]^ According to the higher *R* ^2^ value of 0.95, the *f*_c_ ≈43 wt.% is reliable, thereby the content of BaTiO_3_ around *f*_c_ for BaTiO_3_‐PVDF composites shows the highest *Q* value, which can be considered as ideal dielectrics to obtain epsilon‐negative response.

In contrast with BaTiO_3_‐PVDF composites, composites with a variety of AgNWs content display absolute frequency dispersion as shown in Figure 2c,d. For *x* wt% AgNWs‐(BT‐PVDF) composites, when the value of *x* is equal to 0 and 1, the value of Ac conductivity is below 10^−5^ S cm^−1^ at 10 kHz, resulting in the insulating properties for those composites. Ac conductivity increases linearly with increasing frequency, indicating hopping conduction behavior.^[^ ^18^ ^]^ When the AgNWs content is 2 wt%, the Ac conductivity is close to 1.5 wt% AgNWs‐(BT‐PVDF) composites, resulting in a significant difference between experimental data and theory. In this transitional regime near the *f*_c_, the relationship between electrical conductivity and filler content exhibits a nonlinear deviation from percolation theory predictions, leading to substantial discrepancies between experimental measurements and theoretical fitting curves. Significantly, when the AgNW concentration substantially surpasses *f*_c_, the conductivity behavior conforms well to the percolation model, showing excellent agreement with the theoretical fitting curve. The Ac conductivity demonstrates remarkable enhancement, increasing from 1.6 × 10^−^^3^ S cm^−1^ at *x* = 3 to 2.1 × 10^−^^3^ S cm^−1^ (0.21 × 10^−^^2^ S cm^−1^), and further reaching 0.59 S cm^−1^ at *x *= 4. Moreover, the Ac conductivity mechanism remains nearly constant across frequencies, indicating a typical metallic characteristic.^[^ ^25^ ^]^ Similarly, Ac conduction behavior is directly related to the percolation phenomenon.^[^ ^26^ ^]^ It should be noted that, the frequency dispersion Dc conductivity obeys percolation theory as shown in Figure 2d. We can conclude that the Ac conductivity behavior of AgNWs‐(BT‐PVDF) composites is a transition from hopping conduction to metallic conduction.^[^ ^26^, ^27^ ^]^

**Figure 3:**
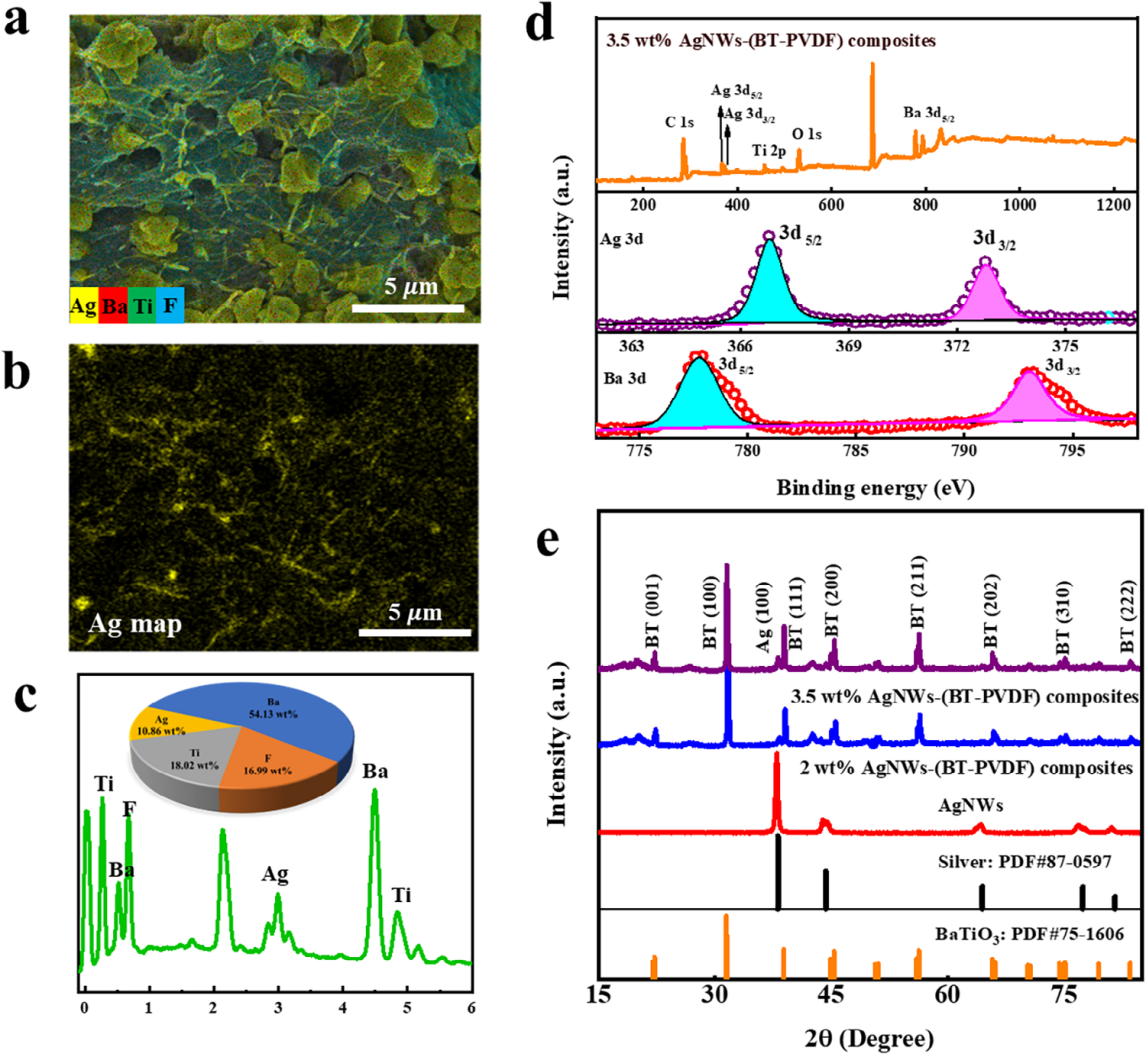
SEM image a) and Ag map b) and EDS spectra c) of 1.5 wt% AgNWs‐(BT‐PVDF) composites, XPS d) and XRD e) patterns of AgNWs and AgNWs‐(BT‐PVDF) composites.

As shown in **Figure** **3**a–c, we can clearly see the distribution of AgNWs and BaTiO_3_ in the PVDF matrix. When AgNWs content increased to 1.5 wt%, AgNWs are evenly distributed in the composites, leading to a dense envelop on BaTiO_3_ and PVDF surface. And then, 3D percolation AgNWs networks are constructed that can provide pathways for the free electrons’ transportation in composites.^[^ ^28^ ^]^ Figure 3d,e reveals the XRD and XPS patterns of BaTiO_3_, AgNWs, and their composites with varying content of AgNWs. The high‐resolution XPS spectra of Ag 3d and Ba 3d of 3.5 wt.% AgNWs‐(BT‐PVDF) composites are exhibited in Figure 3d, doublet featuring signals of 3d_5/2_ and 3d_3/2_ ≈777.7 and 793.1 eV are associated with BaTiO_3_. Observed peaks at 366.7 and 372.7 eV correspond to Ag 3d_5/2_ and Ag 3d_3/2_, peaks at 6 eV spin‐orbit splitting indicate highly crystalline AgNWs.^[^ ^29^ ^]^ Furthermore, XRD patterns confirm the phase of BaTiO_3_ and AgNWs as shown in Figure 3e. Characteristic peaks at circa 22.2°, 31.5°, 39.0°, 45.5°, 56.2°, 65.9°, 75.2°, and 83.5° match well with standard BaTiO_3_ JCPDS card PDF#75‐1606, corresponding to (001), (100), (111), (200), (211), (202), (310), and (222) planes.^[^ ^30^ ^]^ Similarly, diffraction peaks ≈38.4°, 44.3°, 64.5°, 77.5°, and 81.1° indicate a face‐centered cubic structure, consistent with the (100), (200), (220), (311), and (222) planes, which align with the standard silver JCPDS card #87‐0597.^[^ ^14^ ^]^

**Figure 4:**
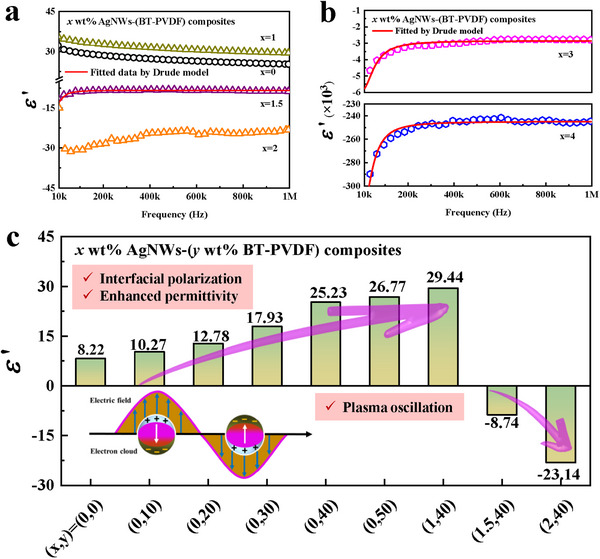
Frequency dispersion of dielectric constant a, b) and dielectric constant at 1 MHz of *x* wt% AgNWs‐(*y* wt% BT‐PVDF) composites c), the insert in (c) is the schematic illustration of the plasma oscillation model.

As discussed above, 40 wt% BaTiO_3_‐PVDF composites as matrix and AgNWs as conducting fillers are explored to fabricate epsilon‐negative composites. AgNWs are of particular interest due to their high aspect ratios, easy processability, which have significant applicability in the state‐of‐the‐art field, especially for generating plasma oscillation.^[^ ^31^, ^32^ ^]^ Therefore, dielectric performances consisting of permittivity and Ac conductivity of 40 wt% BaTiO_3_‐PVDF composites with varying AgNWs content are exploited in this section. In **Figure** **4**a,b, there are two noteworthy features on dielectric constant performance. Compared with 40 wt% BaTiO_3_‐PVDF composites, the value of dielectric constant of 1 wt% AgNWs‐(BT‐PVDF) composites is remarkably enhanced due to AgNWs’ strong interfacial polarization ability. In general, AgNWs acting as a series of micro‐capacitors accumulate interface charges and subsequently generate interface polarization, thereby enhancing the dielectric constant. A slight decrease in permittivity is attributed to dielectric relaxation behavior and the Maxwell‐Wagner‐Sillars effect, both contributing to the overall dielectric relaxation.^[^ ^33^ ^]^ When the content of AgNWs exceeds 1.5 wt%, the dielectric constant transforms from positive to negative. Specifically, those values become more negative with increasing AgNWs content, which is attributed to the strong carrier concentration and carrier mobility.^[^ ^34^ ^]^ The Drude model is commonly used to describe the onset of negative permittivity for a better understanding.^[^ ^35^ ^]^ The equations are as follows:

(1) $$\varepsilon^{\prime }=\varepsilon_{\infty }-\frac{\omega_{p}^{2}}{\omega^{2}+i\gamma \omega }$$

(2) $$\omega_{p}=\sqrt{\frac{Ne^{2}}{m_{e}^{*}\varepsilon_{0}}}$$

where ɛ_∞_ is high‐frequency permittivity, γ is the electron damping term, ω_p_ and ω is the plasma frequency and angular frequency, *N*, and $m_{e}^{*}$ is the free‐electron volume density and effective mass of the electron. Fitting curves along with the experimental data of *x* wt% AgNWs‐(BT‐PVDF) composites (*x *= 1.5, 3, and 4) are shown in Figure 4a,b. When the AgNWs content is 1.5 wt.% or 2 wt.%, the absolute value of negative permittivity remains relatively small, ranging between −10 and −30. This phenomenon, accompanied by lower frequency dispersion, can be attributed to the moderate effective carrier concentration. In contrast, when the AgNWs content increases to 3 wt% or 4 wt%, the enhanced carrier concentration leads to both a higher plasma frequency and a reduced electron damping factor. Consequently, the epsilon‐negative behavior exhibits more pronounced frequency dispersion, which conforms to the characteristic Drude model. The reliability factor *R* ^2^ and plasma frequency (ω_p_) are calculated, and the obtained *R* ^2^ values exceed 0.90, indicating good agreement with experimental data.^[^ ^19^ ^]^ When the AgNWs content is 1.5 wt.%, the ω_p_ and γ are measured as 4.06 × 10⁵ and 1.84 × 10⁵rad s^−1^, respectively. As the AgNWs content increases, ω_p_ exhibits a significant enhancement, reaching 3.18 × 10⁶ rad s^−1^ for *x *= 3 and 1.31 × 10⁷ rad s^−1^ for *x *= 4. This trend can be attributed to the increased effective carrier concentration in the composite. In contrast, the damping constant γ decreases with higher AgNWs content, dropping to 5.78 × 10⁴ rad s^−1^ for *x *= 3 and further to 4.66 × 10⁴ rad s^−1^ for *x *= 4. Notably, since γ remains much smaller than ω_p_ across all compositions, the epsilon‐negative behavior of the material is primarily governed by ω_p_.

Reportedly, laminated structure composites are theoretically equivalent to capacitive circuit networks with three series‐connected capacitors. When the equivalent capacitor of the middle layer with epsilon‐negative behavior is comparable to the outer layers, then the capacitance of the laminated structure composites will become infinite. Consequently, 1.5 wt.% AgNWs‐(BT‐PVDF) composites with small absolute values of dielectric constant are expected ideal candidate in achieving comparable capacitance.^[^ ^36^ ^]^ Dielectric constant of *x* wt% AgNWs‐(*y* wt% BT‐PVDF) composites, consisting of positive and negative values at 1 MHz, is depicted in Figure 4c. The enhancement of positive dielectric constant value from 8.22 to 29.44 is attributed to interfacial polarization, and the negative dielectric constant value of −8.74 is due to plasma oscillation stemming from conductive networks.^[^ ^37^ ^]^

**Figure 5:**
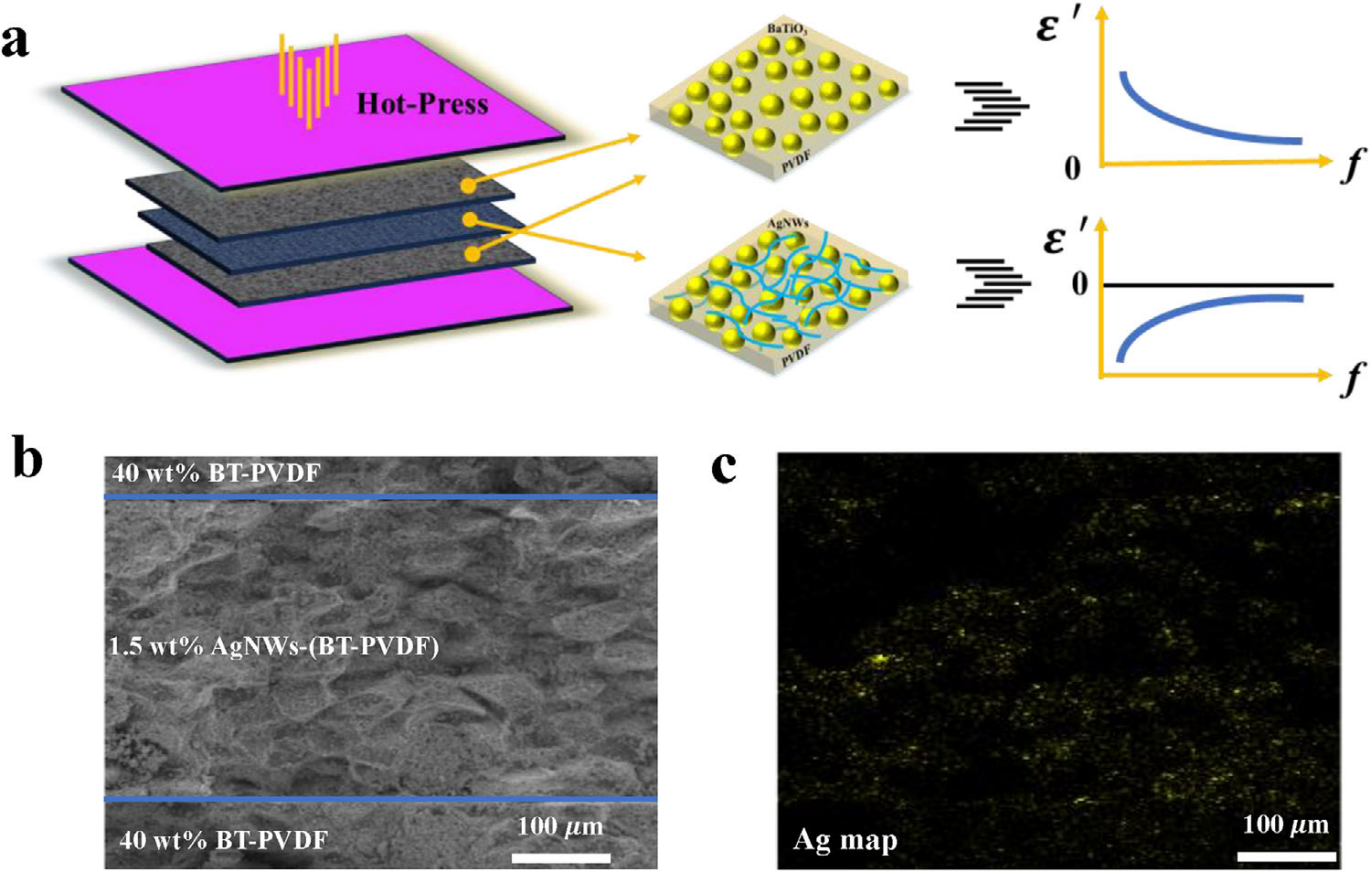
Schematic illustration of laminated structure composites, consisting of epsilon‐positive and epsilon‐negative layers a), cross‐sectional SEM image b), and Ag map c) of A‐B‐A laminated structure composites.

**Figure** **5** shows the schematic illustration and microstructures of A‐B‐A laminated structure composites, where outer layers A are epsilon‐positive composites (*x* wt% BaTiO_3_‐PVDF) and middle layer B is epsilon‐negative composites (*x* wt% AgNWs‐(BT‐PVDF)), and then A‐B‐A laminated structure *x* wt% BaTiO_3_‐PVDF/*x* wt% AgNWs‐(BT‐PVDF)/*x* wt% BaTiO_3_‐PVDF is described as *x* BT‐PVDF/AgNWs/*x* BT‐PVDF. In particular, the epsilon‐positive BaTiO_3_‐PVDF composites exhibit typical dielectric relaxation behavior with the increase of frequency, while epsilon‐negative AgNWs‐(BT‐PVDF) composites show the Drude‐type plasma oscillation behavior.^[^ ^3^, ^9^ ^]^ When the absolute dielectric constant value of epsilon‐negative composites approaches that of epsilon‐positive composites, then the capacitance of laminated structure composites will be infinite. Therefore, laminated structure composites with epsilon‐negative layer are of crucial significance in dielectrics, and those materials are also called meta‐capacitors. Figure 5b,c are the SEM image and Ag map of A‐B‐A laminated structure composites, layers with different content of BaTiO_3_ and AgNWs are clearly observed, and suggest the three layers are merged into a whole composite. At the same time, the thickness and composition of the composites can be tailored during the fabrication process; the dielectric performances will be explored in the next section.

**Figure 6:**
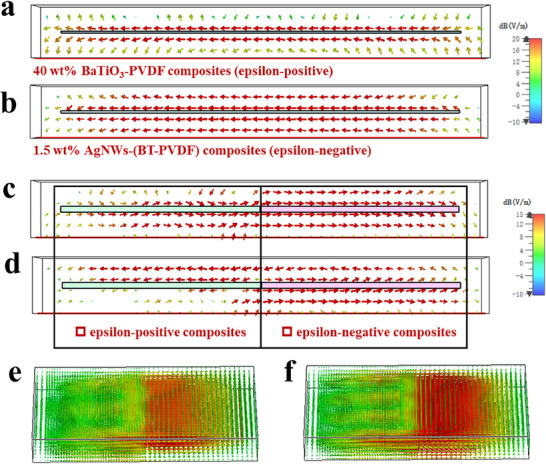
Distribution of electric field intensity a, c (left, at 400 kHz), d (left, at 800 kHz) and e) and b, c (right, at 400 kHz), d (right, at 800 kHz), f) for 40 wt% BaTiO_3_‐PVDF composites and 1.5 wt% AgNWs‐(BT‐PVDF) composites, c‐d show the cross‐section view of composites.

To investigate the local field enhancement effect of epsilon‐negative materials in laminated structure meta‐capacitors, simulation models for both epsilon‐negative materials and conventional epsilon‐positive materials have been constructed using the CST Studio Suite. The distribution of electric field intensity is depicted in **Figure** **6**. From the Figure 6a,b, the 1.5 wt% AgNWs‐(BT‐PVDF) composites have a stronger intensity of electric field than 40 wt% BaTiO_3_‐PVDF composites, which is attributed to the near‐zero dielectric constant. Theoretically, when the dielectric constant approaches zero, the material exhibits remarkable capabilities for electromagnetic manipulation, including supercoupling and localized electric field enhancement. These effects are advantageous for boosting the effective dielectric constant of laminated structure meta‐capacitors.^[^ ^38^, ^39^ ^]^ As shown in Figure 6c–f, when we connect the epsilon‐negative and epsilon‐positive layers together, we observe that as the frequency increases from 400 kHz to 800 kHz, 1.5 wt% AgNWs‐(BT‐PVDF) composites display a “magnetic‐like” effect. This effect includes strong absorption of the electric field at the boundary edge, demonstrating a significant local field enhancement.^[^ ^40^, ^41^ ^]^ Furthermore, by examining the 3D electric field distribution diagram, it becomes evident that epsilon‐negative composites possess a robust ability to manipulate electric field distribution. This capability facilitates strong interface polarization at the interface of laminated structure composites, subsequently enhancing the dielectric performance of meta‐capacitors.

**Figure 7:**
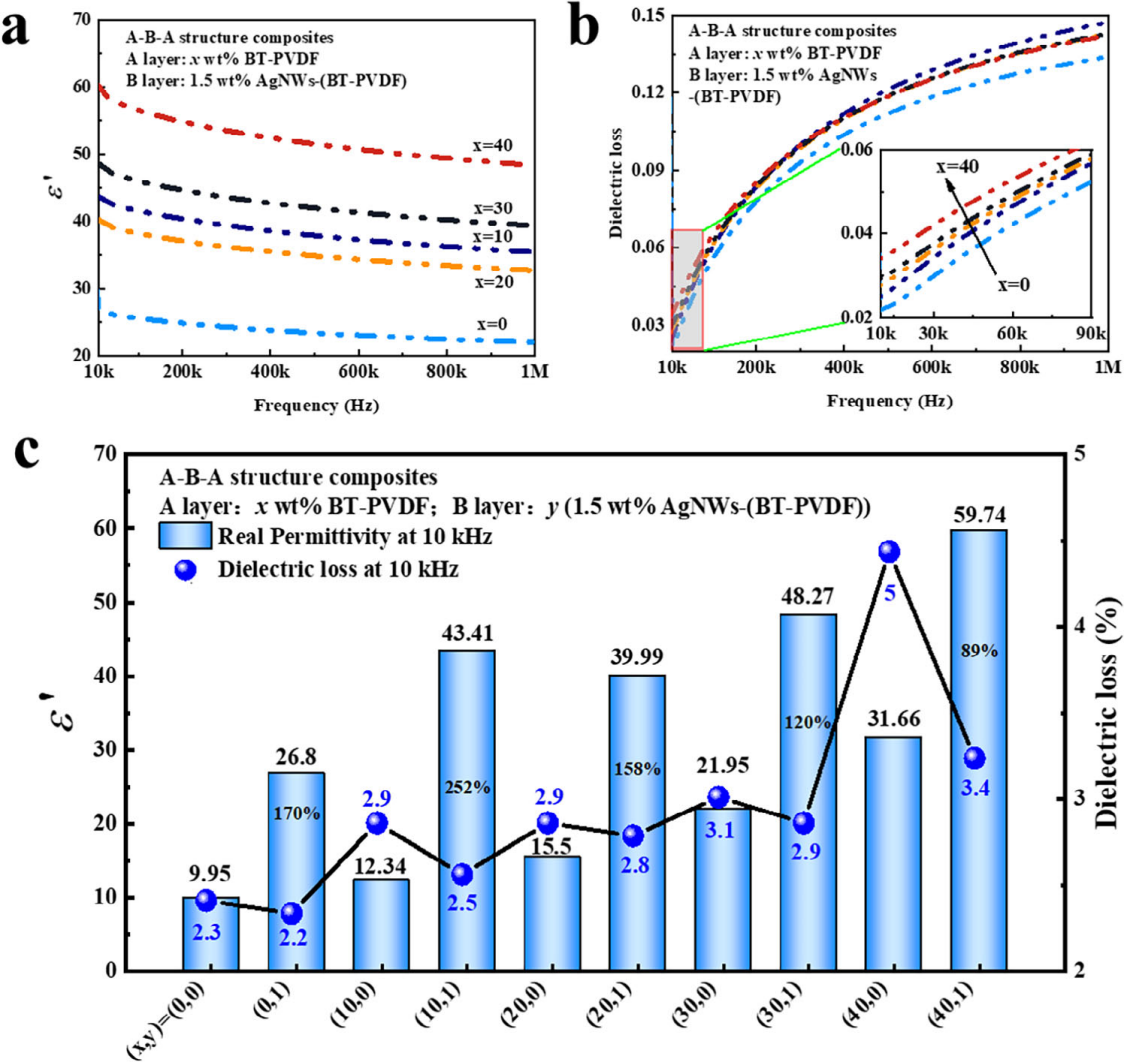
Frequency dependence of dielectric constant a), dielectric losses b), a comparison of properties between composites with or without epsilon‐negative layer c), The inset in (b) provides an enlarged view of dielectric losses.

**Figure** **7**. provides a comprehensive investigation of dielectric properties, including dielectric constant and losses of the laminated structure meta‐capacitors. It should be noted that composites with epsilon‐negative layer demonstrate an enhancement of dielectric constant, as depicted in Figure 7a. The value of the dielectric constant monotonically increases with the increase of BaTiO_3_ content in the outer layers. For instance, the dielectric constant increases from 9.95 of pristine PVDF to 31.66 of P‐40 wt.% BaTiO_3_‐PVDF composites, and further increases to 59.74 of 40 wt.% BT‐PVDF/AgNWs/40 wt.% BT‐PVDF composites. These values represent increments of 189% and 600% over 40 wt.% BaTiO_3_‐PVDF composites and pure PVDF, respectively. The enhancement of the dielectric constant in a layered structure composite can be attributed to remarkable plasma oscillations derived from the middle epsilon‐negative layer.^[^ ^42^ ^]^ Epsilon‐negative composites lead to an infinite overall capacitance in meta‐capacitors, resulting in a high dielectric constant. The dielectric constant of AgNWs‐(BT‐PVDF) composites is relatively high at low frequencies but decreases with increasing frequency, a behavior attributable to dielectric relaxation. The elevated dielectric constant at low frequencies primarily arises from interfacial polarization effects. In this system, blocking electrodes inhibit the movement of ions from the electrolyte into the external circuit, causing them to accumulate at the interface. This ion accumulation induces polarization, thereby enhancing the dielectric constant. However, at higher frequencies, the electric field direction changes too rapidly for the ions to realign effectively, resulting in a diminished interfacial polarization effect and, consequently, a lower dielectric constant.^[^ ^43^, ^44^ ^]^

Interestingly, those composites still maintain lower dielectric losses, as illustrated in Figure 7b. Across the tested frequencies, dielectric losses consistently remain below 0.15, proving that BaTiO_3_‐PVDF composites can effectively restrict electron transfer. Meanwhile, dielectric losses of laminated structure composites increase with increasing BaTiO_3_ content, which is attributed to the polarization losses. In fact, compared to dielectric losses of BaTiO_3_‐PVDF and AgNWs‐(BT‐PVDF) composites as shown in Figure S1 (Supporting Information), the dielectric losses of meta‐capacitors have been partially mitigated through the implementation of a laminated structure. For example, when the content of BaTiO_3_ of BaTiO_3_‐PVDF composites enhances from 0 to 40, the dielectric losses increase slightly from 0.023 to 0.034 at 10 kHz, which is still acceptable for practical applications, including novel capacitors and energy storage systems.^[^ ^13^, ^42^ ^]^ While the composite's dielectric losses are higher than those of pristine PVDF (e.g., increasing from 0.023 to 0.034 at 10 kHz), they are still acceptable for practical applications. Consequently, these composites strike a balance between dielectric constant and losses, where enhancement of dielectric constant is attributed to the strong polarization ability of the middle epsilon‐negative layer, and lower losses are associated with the outer epsilon‐positive layers. Figure 7c shows a comparison of the dielectric constant and losses at 10 kHz of the composite with or without an epsilon‐negative layer. The value of the dielectric constant can be enhanced 2 times, while the dielectric loss values remain relatively similar for the outer layer. When *x* is between 0 and 40, outer layer BaTiO_3_‐PVDF composites can effectively reduce leakage currents and conduction losses, resulting in the lower dielectric losses of 0.05 at 10 kHz.

**Figure 8:**
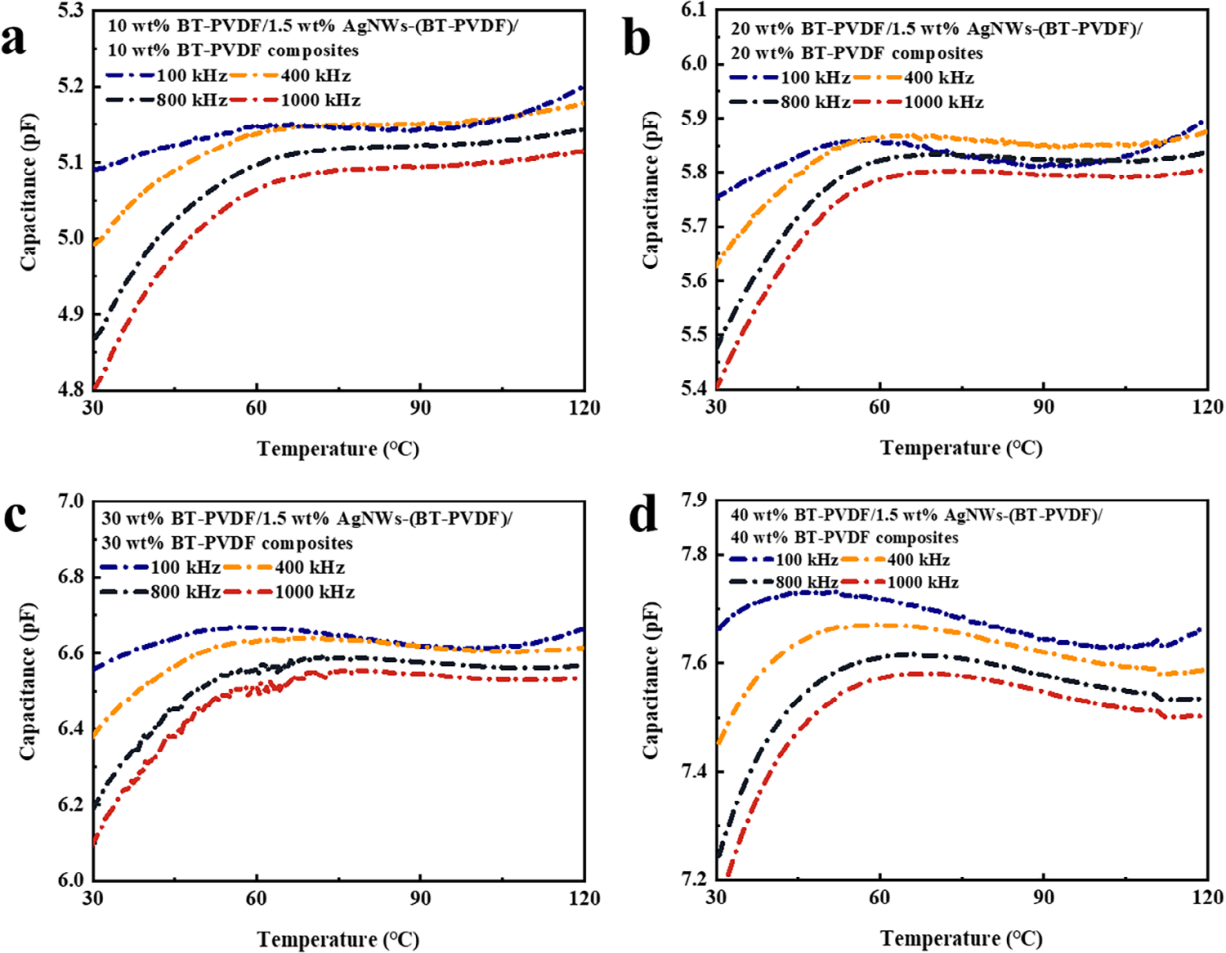
Temperature dispersion of capacitance for meta‐capacitors with different systems of BaTiO_3_‐PVDF composites.

Capacitance‐temperature dispersion of BT‐PVDF/AgNWs/BT‐PVDF composites is depicted in **Figure** **8**. Due to the dielectric relaxation, capacitance values decrease between 30 and 120 °C as frequency increases from 100 kHz to 1000 kHz, and maximum capacitance behavior is observed at 100 kHz.^[^ ^45^ ^]^ With an increase in the BaTiO_3_ content of the outer layer, maxima capacitance values (5.11, 5.90, 6.67, and 7.73) corresponding to different *x* values (10, 20, 30, 40) are observed at varying temperatures (120, 120, 57.3, and 49.6 °C), respectively. The value of capacitance is approaching a constant ≈60–120 °C, indicating a good stability of capacitance‐temperature. For instance, the temperature at which the highest capacitance value occurs at 49.6 °C for 40 BT‐PVDF/AgNWs/40 BT‐PVDF composites, which can be considered the accurate Curie temperature, and the trend of capacitance from temperature‐dependent to temperature‐independent is related to the phase transition between paraelectric and ferroelectric.^[^ ^46^ ^]^

**Figure 9:**
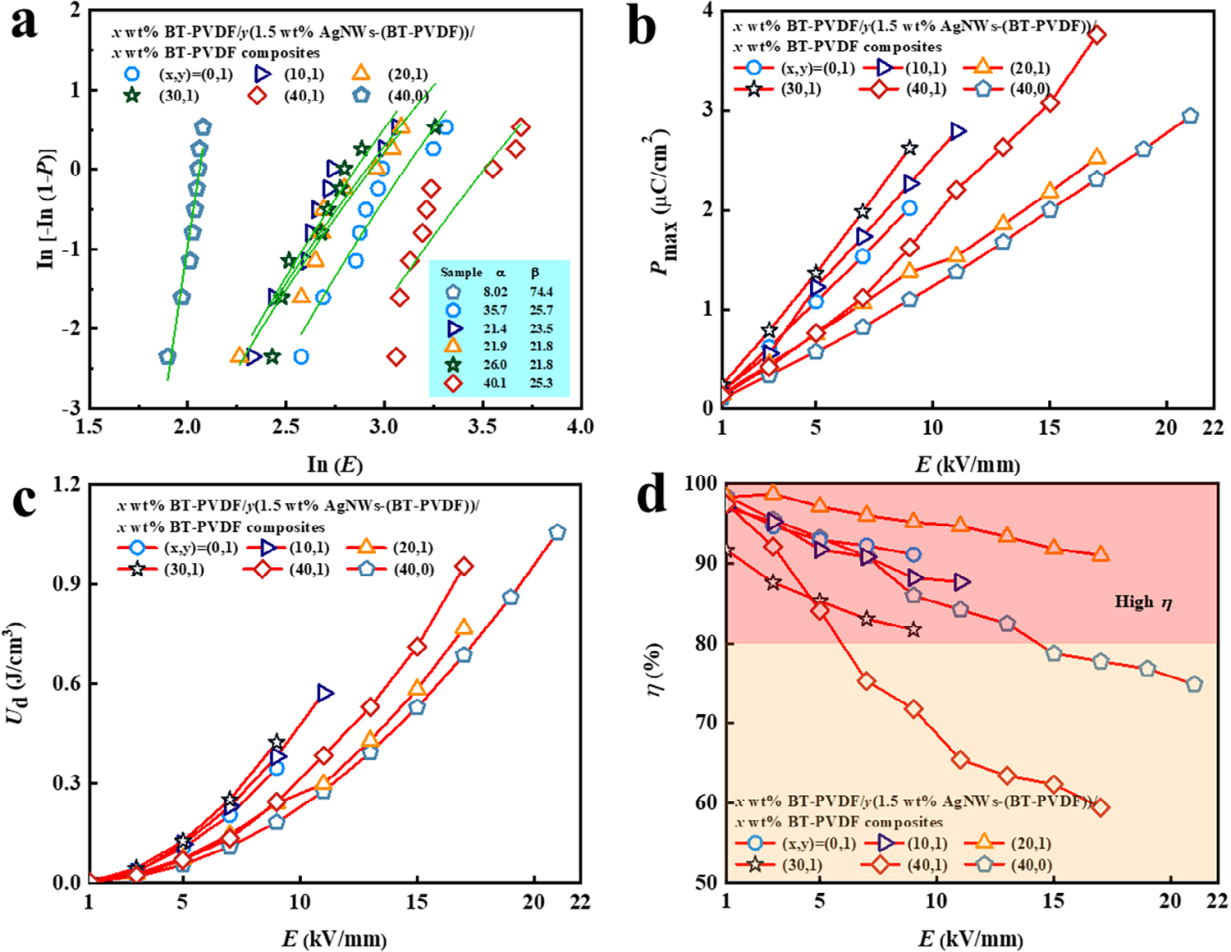
Two‐parameter Weibull distribution plots a), maximum polarization (*P*_max_) b), discharged energy densities (*U*_d_) c) and efficiency (*η*) d) of the *x* wt% BaTiO_3_‐PVDF/1.5 wt.% AgNWs‐(BT‐PVDF)/*x* wt% BaTiO_3_‐PVDF meta‐capacitors.

In order to study the influence of the epsilon‐negative response in the meta‐capacitors on their energy storage performance, the Weibull breakdown strength (*E*_b_), maximum polarization (*P*_max_), discharge energy densities (*U*_d_), and efficiency (η) are comprehensively investigated in this section. The two‐parameter Weibull distribution plot shows the distribution of *E*_b_ in the composites. The distribution can be described by the Weibull distribution Equation (3),^[^ ^47^ ^]^

(3) $$P(E)=1-\exp\;\;\left[-{\left(\frac{E}{a}\right)}^{\beta }\right]$$

where *P*(E) represents the cumulative failure probability, *E* is the experimental breakdown electric field, α is the electric field, and β is the shape parameter reflecting the scatter. To suppress losses, laminated structure *x* wt% BT‐PVDF/AgNWs/*x* wt% BT‐PVDF meta‐capacitors exhibit increased α values with the BaTiO_3_ content increasing in **Figure** **9** **a**. For example, the α of the laminated structure composites improves from 8.02 to 40.1 kV mm^−1^, which should be attributed to the high insulating property of the *x* wt% BaTiO_3_‐PVDF layer, blocking the growth of breakdown paths. However, when BaTiO_3_ content exceeds critical points (e.g., *x *= 10), the positive contribution of defects starts to exceed the negative contribution of BaTiO_3_, leading to a deterioration in α. Besides, the highest β value of 74.4 of PVDF/AgNWs/PVDF composites indicates excellent dielectric reliability.^[^ ^48^ ^]^ The variations of *P*_max_ and remnant polarization (*P*_r_) under external electric field are depicted, as derived from the *P*‐*E* loops (see Figure 9b; Figure S2a, Supporting Information). Compared with 40 wt.% BaTiO_3_‐PVDF composites, laminated structure composites with epsilon‐negative layer exhibit higher *P*_max_ and lower *P*_r_, which is conducive to improving energy density and efficiency.^[^ ^49^ ^]^ For example, *P*_max_ and *P*_r_ values of 30 wt.% BT‐PVDF/AgNWs/30 wt.% BT‐PVDF composites are 2.6 and 0.43 µC cm^−2^, while *P*_max_ and *P*_r_ values of 40 wt.% BaTiO_3_‐PVDF are 1.09 and 0.21 µC cm^−2^. Therefore, a significant enhancement of (*P*_max_‐*P*_r_) is related to the interfacial polarization between epsilon‐negative and epsilon‐positive layers.

Energy storage performances are evaluated in terms of stored energy density (*U*_s_) and *U*_d_, along with the η (*U*_d_ */U*_s_). Energy storage capabilities of *U*_s_, *U*_d_, and η are illustrated in Figure 9c,d and Figure S2b (Supporting Information). A lower *E*_b_ value of 9 kV mm^−1^, laminated structure 30 wt.% BT‐PVDF/AgNWs/30 wt.% BT‐PVDF composites exhibit the highest *U*_s_ of 0.52 J/cm^3^ and *U*_d_ of 0.42 J cm^−3^, which are 148% and 133% greater than those of a single layer 40 wt.% BaTiO_3_‐PVDF composites (*U*_s_ of 0.21 J cm^−3^ and *U*_d_ of 0.18 J cm^−3^). In essence, the increase of *U*_s_ can be attributed to the enhanced permittivity and breakdown strength, in which the enhanced permittivity was ascribed to the interfacial polarization and local field enhancement effect derived from the epsilon‐negative layer, and breakdown strength can be improved by blocking the propagation of electric trees in the laminated structure meta‐capacitors. In general, the incorporation of an epsilon‐negative layer as the middle layer within novel multi‐layer composites, flanked by epsilon‐positive layers on the outer sides, enables the simultaneous enhancement of energy storage density. For a higher electric field strength of 17 kV mm^−1^, 20 wt.% BT‐PVDF/AgNWs/20 wt.% BT‐PVDF composites present a higher *U*_s_ of 0.77 J cm^−3^ and a higher *η* of 91%, which are 12% and 17% greater than those of single‐layer 40 wt.% BaTiO_3_‐PVDF composites.^[^ ^50^ ^]^ As shown in Figure 9d, the *η* value of 40 wt.% BaTiO_3_‐PVDF composites is sharply deteriorated under the increasing electric field. In contrast, 20 wt.% BT‐PVDF/AgNWs/20 wt.% BT‐PVDF composites with epsilon‐negative layer result in an extremely high *η* of 91%, surpassing the *η* value of 78% of 40 wt.% BaTiO_3_‐PVDF composites. While the incorporation of an epsilon‐negative layer leads to a modest decrease in α, the resulting laminated composite structure demonstrates substantial improvements in both energy storage density and energy conversion efficiency.^[^ ^51^, ^52^ ^]^ In conclusion, the investigation of *U*_s_, *U*_d_, and η emphasizes that meta‐capacitors with an epsilon‐negative layer exhibit remarkable improvements in energy storage capabilities, making them highly promising for the next generation of capacitors.

## 3 Conclusion

In this study, the concept of a laminated structure meta‐capacitor composed of epsilon‐negative and epsilon‐positive layers was pioneered. The strategic modulation of laminated meta‐capacitors opened a novel design for enhancing dielectric performance and energy storage density. Combined with simulation of electric field intensity, the strong interfacial polarization and local field enhancement effects of epsilon‐negative materials were demonstrated comprehensively, where the dielectric constant of meta‐capacitors to 59.74. This represents an improvement of 189% over 40 wt.% BaTiO_3_‐PVDF composites, which have a dielectric constant of 31.66, and a 600% increase over pristine PVDF nanocomposites, which have a dielectric constant of 9.95 at 10 kHz. Additionally, by incorporating insulating BaTiO_3_‐PVDF outer layers, the leakage current and conduction losses in meta‐capacitors were effectively reduced to 0.05 at 10 kHz. This design also achieved excellent energy density properties due to the synergistic effects between the epsilon‐negative and epsilon‐positive layers, reaching a high energy density of 0.77 J cm^−^^3^ and an impressive efficiency of 91% in the 20 wt.% BT‐PVDF/AgNWs/20 wt.% BT‐PVDF composites. Overall, this work highlights the potential of laminated structure meta‐capacitors with an epsilon‐negative layer to enhance dielectric properties and energy storage performances, positioning them as promising candidates for next‐generation capacitors.

## 4 Experimental Section

### Raw Materials

PVDF with a molecular weight of 100 000 was sourced from Dongguan Zhanyang Polymer Material Co., Ltd (China). BaTiO_3_ was purchased from Nanjing Suzhan Intelligent Technology Co., Ltd (China). AgNWs with high aspect ratios were synthesized by a polyol method.^[^ ^14^ ^]^ Absolute ethanol with a purity of at least 95% was supplied by Sinopharm Chemical Reagent Co., Ltd (China). All the raw materials and chemicals were used as received without undergoing further purification.

### Preparation Laminated Meta‐Capacitors

Single‐layer and laminated structure composites were prepared using a hot‐press strategy, and the preparation involved dispersing the raw materials, PVDF, BaTiO_3_ (BT), and AgNWs in absolute ethanol and stirring them at room temperature until a uniform state was achieved. After magnetic stirring, the mixture was dried in an oven at 100 °C and then shaped into thin disks with a diameter of 20 mm and a thickness of ≈1 mm. For the single‐layer composites, a homogeneous mixture of PVDF and BaTiO₃ was hot‐pressed at 125 °C under 25 MPa for 3 min. The laminated composites were prepared through a layer‐by‐layer assembly process, where the first BaTiO₃‐PVDF layer was initially formed at 125 °C under 15 MPa for 30 s. Subsequently, the second AgNWs‐(BT‐PVDF) layer was deposited and consolidated under identical conditions. The third layer was similarly prepared using the above method, and the pressure and hold time were increased to 25 MPa and 3 min. Finally, a well‐integrated three‐layer laminated structure can be obtained, respectively. The succinct symbol of “*x*” of *x* wt% BaTiO_3_‐PVDF and *x* wt% AgNWs‐(BaTiO_3_‐PVDF) represented the mass fraction of BaTiO_3_ and AgNWs. For the “A‐B‐A” laminated structure composites, symbols of A and B represented *x* wt% BaTiO_3_‐PVDF and *x* wt% AgNWs‐(BaTiO_3_‐PVDF) composites.

### Characterization and Dielectric Measurement

The crystallinity of AgNWs, BaTiO_3_‐PVDF, and AgNWs‐(BaTiO_3_‐PVDF) was characterized using X‐ray diffraction (XRD) with a PANalytical X'Pert PRO instrument from the Netherlands. Morphologies of laminated structure composites were studied by field emission scanning electron microscopy (FESEM) with a JSM 7500F instrument from Japan. Element composition and chemical valence were examined by X‐ray photoelectron spectroscopy (XPS) with Thermo ESCALAB 250XI from the USA. For dielectric measurement, all of the samples were coated with a thin layer of gold to reduce surface resistance and then measured using an LCR meter (Keysight, E4980AL, USA). Dielectric properties were calculated using equations from the reference.^[^ ^53^ ^]^ Breakdown strength was determined using a setup equipped with a Trek 609A amplifier, provided by PolyK Technologies from the USA. Energy storage performances, including energy density and efficiency, were demonstrated by PE hysteresis loops, which were collected using a ferroelectric test system from PolyK Technologies in the USA.

## Conflict of Interest

The authors declare no conflict of interest.

## Author Contributions

Z.W. performed methodology, validation, formal analysis, investigation, data curation, and wrote the original draft. K.S. performed methodology, validation, formal analysis, investigation, and data curation. Y.Y. performed methodology, validation, formal analysis, investigation, and funding acquisition. X.Y. and Q.H. performed data curation, validation, formal analysis, and investigation. C.S. and R.F. reviewed, supervised, and performed project administration and funding acquisition.

## Supporting information



Supporting Information

## Data Availability

The data that support the findings of this study are available from the corresponding author upon reasonable request.
