# Supplementary material for: Silver Nanowires Epsilon‐Negative Metacomposites in Constructing Laminated Structure Meta‐Capacitors
Source: Small. 2025 Jun 30;21(46):2501848. doi: 10.1002/smll.202501848 (PMC12632440; doi:10.1002/smll.202501848)
Supplement: Supplementary file 1 — Supporting Information [file SMLL-21-2501848-s001.docx]

**SILVER NANOWIRES EPSILON-NEGATIVE METACOMPOSITES IN CONSTRUCTING LAMINATED STRUCTURE META-CAPACITORS**

Zongxiang Wang^1^^, 4^, Kai Sun^2, 4 *^, Yuan Yuan^1, 4*^, Xin Yao^2^, Qing Hou^1, 4^, Chaoyun Song^3*^, Runhua Fan^1, 4*^

^1^ Logistics Engineering College, Shanghai Maritime University,

Shanghai 201306, China

^2^ College of Ocean Science and Engineering, Shanghai Maritime University,

Shanghai 201306, China

^3^ Department of Engineering, Faculty of Natural, Mathematical & Engineering Sciences, King's College London, London, WC2R 2LS, UK

^4^ Shandong Key Laboratory of Metamaterial and Electromagnetic Manipulation Technology, Jinan 250061, China

* Corresponding authors

E-mail addresses:

[kais@shmtu.edu.cn](mailto:kais@shmtu.edu.cn), [yuanyuan@shmtu.edu.cn](mailto:yuanyuan@shmtu.edu.cn), [chaoyun.song@kcl.ac.uk](mailto:chaoyun.song@kcl.ac.uk), rhfan@shmtu.edu.cn

****The dielectric loss spectra of BaTiO_3_-PVDF (BT-PVDF) and AgNWs-(BT-PVDF) composites are presented in Fig. S1. For the epsilon-positive BT-PVDF layers, the dielectric loss remains relatively low (0.03-0.18) across the entire measured frequency range, suggesting that polarization losses constitute the primary contribution to the total dielectric loss. In contrast, the AgNWs-(BT-PVDF) composites exhibiting epsilon-negative behavior demonstrate significantly higher dielectric losses compared to their epsilon-positive counterparts. This marked increase in dielectric loss can be attributed to the formation of conductive Ag networks within the composite, which introduces substantial conduction losses to the system.

**Fig. S1.** Dilectric loss of BaTiO_3_-PVDF (a) and AgNWs-(BT-PVDF) composites

**Fig. S2.** Remnant polarization (*P*_r_) (a) and stored energy density (*U*_s_) (b) of BaTiO_3_-PVDF/AgNWs-(BT-PVDF)/BaTiO_3_-PVDF composites

**Fig S2a** shows the variations of *P*_r_ of the BaTiO_3_-PVDF/AgNWs-(BT-PVDF)/BaTiO_3_-PVDF composites with external electric field. It can be seen that 20 wt% BaTiO_3_-PVDF/AgNWs-(BT-PVDF)/20 wt% BaTiO_3_-PVDF composites exhibits lowest *P*_r_ values, which is beneficial to improving of energy density and efficiency. Meanwhile, the enhancement of *P*_r_ is mainly attributed to the higher conductive losses, which is derived from the percolation networks [1]. **Fig. S2b** presents the store energy density of the composite that is derived from the P-E loops by integration of the area between the charge curve and the ordinate. The value of *U*_s_ of the single layer 40 wt% BaTiO_3_-PVDF composites is below the sandwich-structured nanocomposites with epsilon-negative layer [2,3]. For instance, at lower breakdown electric field of 9 kV/mm, 30 wt% BaTiO_3_-PVDF/AgNWs-(BT-PVDF)/30 wt% BaTiO_3_-PVDF composites show highest *U*_d_ of 0.42 J/cm^3^, which is 133% greater than that single layer 40 wt% BaTiO_3_-PVDF composites (*U*_d_ of 0.18 J/cm^3^).

**References**

1. Sun, L.; Shi, S.; Wang, H.; Xie, P.; Dastan, D., Optimizing strategy for the dielectric performance of topological-structured polymer nanocomposites by rationally tailoring the spatial distribution of nanofillers, *Eng Sci*. **2020,** *12* (5), 95-105.

2. Feng, Q. K.; Zhong, S. L.; Pei, J. Y.; Zhao, Y.; Zhang, D. L.; Liu, D. F.; Zhang, Y. X.; Dang, Z. M., Recent progress and future prospects on all-organic polymer dielectrics for energy storage capacitors, *Chem Rev* **2022,** *122* (3), 3820-3878.

3. Feng, M.; Feng, Y.; Zhang, T.; Li, J.; Chen, Q.; Chi, Q., Recent advances in multilayer‐structure dielectrics for energy storage application, *Adv Sci*. **2021,** *8*(23), 2102221.
